# Concurrent overexpression of RET/PTC1 and TTF1 confers tumorigenicity to thyrocytes

**DOI:** 10.1530/ERC-13-0310

**Published:** 2013-12

**Authors:** Toyoshi Endo, Tetsuro Kobayashi

**Affiliations:** Third Department of Internal MedicineInterdisciplinary Graduate School of Medicine and Engineering, University of YamanashiChuo City, Yamanashi, 409-3898Japan

**Keywords:** RET/PTC, thyrocytes, thyroid transcription factor-1, tumorigenesis

## Abstract

A variant located on 14q13.3 nearest to thyroid transcription factor-1 (TTF1) predisposes individuals to thyroid cancer, but whether this variant is related to the *RET/PTC* rearrangement associated with human papillary thyroid carcinomas (PTCs) is unknown. The aims of this study were to investigate the effects of RET/PTC1 on the expression of thyroid-specific genes in thyrocytes and their relationship with malignant transformation of the thyrocytes. In the absence or presence of TSH, an extracellular signal-regulated kinase was phosphorylated in FRTL5 cells that stably expressed RET/PTC1, and these cells grew independently of TSH. FRTL (RET/PTC1) cells produced 566% more thyroglobulin mRNA and 474% more Na+/I− symporter mRNA than did the control FRTL (pcDNA) cells. FRTL (RET/PTC1) cells expressed 468% more *Ttf1* mRNA than did FRTL (pcDNA) cells, but these two cell types did not differ significantly with respect to *Pax8* or *Ttf2* mRNA levels. When FRTL (RET/PTC1) cells and FRTL (pcDNA), cells were injected into each of nine nude mice, each mouse developed a single tumor at the site of FRTL (RET/PTC1) cell injection; in contrast, tumor formation never occurred at sites of FRTL (cDNA) cells injection. Tumors resulting from FRTL (RET/PTC1) cells retained ^125^I-uptake activity; moreover, the cells invaded into surrounding skeletal muscle. When overexpression of *Ttf1* in FRTL (RET/PTC1) cells was silenced, the cells completely lost their tumorigenic potential. Exogenous *TTF1* cDNA enhanced the tumorigenicity of BHP18-21v cells, human PTC cells that express RET/PTC1, in nude mice. These results indicated that concurrent overexpression of RET/PTC1 and TTF1 confers tumorigenicity to FRTL5 and BHP18-21v cells in nude mice.

## Introduction

Papillary thyroid carcinomas (PTCs) are the most frequent cancers of the thyroid gland, and they are usually well differentiated given their ability to i) take up iodine, ii) secrete thyroglobulin (TG), and iii) be responsive to thyroid-stimulating hormone (TSH; Nikiforov & Nikiforova 2011).

The *RET/PTC* rearrangement and the *BRAF*^V600E^ point mutation are the two most common genetic alterations associated with PTCs; the prevalence of *RET/PTC* varies from 2.5 to 78% (Zou *et al*. 1994, Nikiforov *et al*. 1997), and the prevalence of *BRAF*^V600E^ varies from 23 to 62% (Xing *et al*. 2005, Fugazzola *et al*. 2006).

Rearrangements of the *RET* gene can cause recombination of sequences encoding the intracellular kinase domain of RET with a heterologous gene and thereby generate a chimeric oncogene that induces RAS-dependent activation and consequent ERK activation (Melillo *et al*. 2005). However, constitutive activation of ERK caused by a *RET/PTC* oncogene may or may not be sufficient to induce all hallmarks of cancer *in vivo*. Santoro *et al*. (1996) found that some *RET/PTC1* transgenic mice developed thyroid tumors, but others developed only thyroid hyperplasia. Knostman *et al*. (2007) reported that doxycycline-induced expression of RET/PTC1 led to ERK phosphorylation in mice that carried a doxycycline-regulated *RET/PTC1* transgene; however, thyroid lesions were not found in any of these mice.

These results indicate that oncoproteins such as RET/PTC activate the MEK/ERK cascade, which then promotes an initial wave of dramatic cell proliferation that, in turn, initiates tumor development, but subsequent development of a solid cancer requires an additional unknown lesion or alteration (Pritchard *et al*. 2007).

Gudmundsson *et al*. (2009) recently conducted a genome-wide association study (GWAS) of thyroid cancer cases; they found that a variant predisposes individuals from European populations to thyroid cancer; this variant is located on 14q13.3 near thyroid transcription factor-1 (TTF1), which is also called *NKX2.1*. TTF1 and PAX8 are master regulators of thyroid-specific gene expression. For example, they regulate *TG*, *NIS* (*SLC5A5*), and *TSHR*; moreover, they play pivotal roles in the development of thyroid glands (Sinclair *et al*. 1990, Silberschmidt *et al*. 2011). Therefore, the GWAS findings may be relevant to the pathogenesis of PTCs. However, whether relationships between the variant at 14q13.3 and the genetic alterations in PTCs (e.g., *RET/PTC* rearrangements) exist is unclear.

To assess whether there are important interactions between the 14q13.3 variant and *RET/PTC* rearrangements, we expressed RET/PTC1 in FRTL5 cells, functional thyroid epithelial cells, and studied the effects of RET/PTC1 on the expression of thyroid-specific genes with a particular focus on the expression of *Ttf1*, *Ttf2*, and *Pax8*, and their relationship with tumorigenicity of the cells. Further, *TTF1* cDNA was introduced into BHP18-21v cells, which are human PTC cells, to examine the effects of TTF1 on tumorigenicity of these cells.

## Materials and methods

### Cells, tissues, and animals

FRTL5 cells (CRL8395, ATCC, Manassas, VA, USA) were cultured in Ham F12 medium that contained 5H (insulin 10 ng/ml, cortisol 0.4 ng/ml, transferrin 5 μg/ml, glycyl-l-histidyl-l-lysine 10 ng/ml, and somatostatin 10 ng/ml) and 5% calf serum with or without 10 mU/ml TSH (Sigma–Aldrich, Inc.) (Endo *et al*. 1990). The cells grew in a TSH-dependent manner and expressed seven thyroid-specific genes – *Ttf1*, *Ttf2*, *Pax8*, *Tg*, thyroid peroxidase (*Tpo*), Na+/I− symporter (*Nis*), and Tsh receptor (*Tshr*). BHP18-21v cells were isolated from BHP18-21 cells, which are human thyroid papillary cancer cells that express RET/PTC1 (Ohta *et al*. 1997); BHP18-21v cells were cultured in RPMI-1640 containing 10% FCS, and they expressed PAX8 but not *TTF1*, *TTF2*, *TG*, *TPO*, *NIS*, or *TSHR*. This expression profile is unique to BHP18-21v cells among BHP cell lines; therefore, the BHP18-21v cells we used were free from contamination. BRL-3A cells (CRL-1442, ATCC) were cultured in Ham F12 medium that contained 5% FCS. A 5-bromo-2′-deoxyuridine (BrdU) incorporation assay (BrdU labeling and detection kit, Roche Diagnostics) was used to monitor cell proliferation. Normal human thyroid tissues were obtained from surgical specimens taken from patients with papillary thyroid cancer; each patient gave written informed consent. Male Balb/c nude mice (aged 12 weeks) were obtained from CLEA Japan, Inc., Tokyo, Japan. Each mouse was specific pathogen free and checked for pathogens once every 2 months. All studies performed were approved by the Animal Research Committee at the University of Yamanashi.

### Plasmid construction and transfection

cDNAs were reverse transcribed from mRNA templates that had been isolated from BHP18-21v cells. Using this cDNA sample as template, *RET/PTC1* cDNAs were PCR amplified with the following primers: sense, 5′-CTCCTCCTCCTTTCCCAGCC-3′, and antisense, 5′-GCTCGGCCAATGTGACGTTCAC-3′. Amplified cDNAs were first ligated into a pCR2 vector (Invitrogen Co.) and then isolated insert cDNA was ligated into the KpnI/NotI site of pcDNA3.1-hygro (Invitrogen Co.). Human *TTF1* cDNAs were cloned from human thyroid carcinoma lambda gt11 cDNA library (HL1009, Clontech Lab., Inc.), and an Eco RI insert that contained the full coding sequence (1.4 kb) was ligated into pcDNA3.1zeo. Plasmid DNA (1 μg) was introduced into FRTL5 or BHP18-21v cells with the Gene Pulser (Gene Pulser Xcell; Bio-Rad) at 250 V-750 μF. Stable transformants were selected by adding 300 μg/ml hygromycin B (Wako Pure Chemicals, Inc., Ltd., Osaka, Japan) or 100 μg/ml Zeocin (Life Technologies Co.) to the culture medium. *TTF1* siRNA was expressed in cells from a pSilencer 4.1-CMV neo construct (Applied Biosystems, Inc.); to generate this *TTF1* siRNA construct, two oligonucleotides – 5′-GATTCACACGACTCCGTTCTCAGTTTCAAGAGAACTGACAACGGAGTCGTGTGCA-3′ and 5′-AGCTTGCACACGACTCCGTTGTCAGTTCTCTTGAAACTGAGAACGGAGTCGTGTG-3′ (Kolla *et al*. 2007) – were annealed and ligated into the BamH1/HindIII site of pSilencer 4.1-CMV neo (Applied Biosystems, Inc.) (pSilencer-TTF1). The construct or the pSilencer 4.1-CMV neo-negative control was introduced into cultured cells. Stable transformants were selected by adding 300 μg/ml geneticin (Sigma–Aldrich, Inc.) to the culture medium.

### Quantitative PCR

The Rotor-Gene Q (Qiagen, Inc.) and 13 TaqMan probes (Applied Biosystems, Inc.) – rat *Tg* (Rn01458686_A1), rat *Nis* (Rn01420249_g1), rat *Tshr* (Rn00563612_A1), rat *Ttf1* (Rn01512482_A1), rat *Ttf2* (*Foxe1*) (Rn00594363_s1), rat *Pax8* (Rn00579743_A1), rat *Gapdh* (Rn01775763_g1), human *TTF1* (Hs00968940_m1), human *TG* (Hs00174974_m1), human thyroid peroxidase (*TPO*) (Hs00892519_m1), human *NIS* (Hs00166567_m1), human *RET* (Hs04259657_s1), and human *GAPDH* (Hs02758991_g1) – were used to perform quantitative PCR. Assays for each gene were carried out in triplicate, and transcript levels of thyroid-specific mRNA were normalized to those of *GAPDH* (human) or *Gapdh* (rat). Expression of *GAPDH* or *Gapdh* from the samples was within ±2 cycle number of threshold (*C*t).

### ^125^I-uptake assay and TSH binding activity

The ^125^I-uptake by FRTL5 cells was measured as described previously (Endo *et al*. 1996). ^125^I scintigraphy of the tumors formed in nude mice was performed by injecting Na^125^I into the peritoneal space (Endo & Kobayashi 2010). Radioactivity was monitored with a BAS2500 image analyzer (Fuji Film Co., Tokyo, Japan). ^125^I-TSH binding activity in each cell population was measured using ^125^I-bovine TSH (Cosmic Co., Tokyo, Japan) and methods described by Mizutori *et al*. (2008).

### Statistical analysis

The Student's *t*-test and one-way ANOVA were used to assess the statistical differences between groups.

## Results

### Effects of RET/PTC1 on the morphology of FRTL5 cells

**Figure 1:**
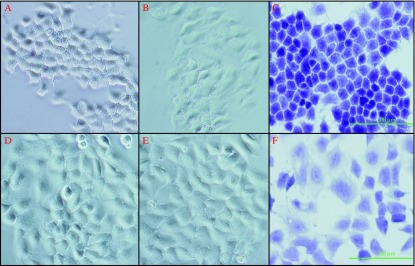
Morphology of FRTL (pcDNA) and FRTL (RET/PTC1) cells. Phase-contrast images of FRTL (pcDNA) cells cultured for 6 days in the presence of 1 mU/ml TSH (A) or absence of TSH (B). Hematoxylin–eosin-stained FRTL (pcDNA) cells cultured in the presence of TSH (C). Phase-contrast images of FRTL (RET/PTC1) cells cultured for 6 days in the presence of 1 mU/ml TSH (D) or absence of TSH (E). Hematoxylin–eosin-stained FRTL (RET/PTC1) cells cultured in the presence of TSH (F). Scale bars, 100 μm.

In the presence of TSH, FRTL5 cells transfected with the empty pcDNAhygro vector (FRTL (pcDNA) cells) were small and round and their cellular borders were well defined (Fig. 1A). When TSH was withdrawn from the medium, these control cells became flattened and the cell borders became obscured (Fig. 1B). We transfected pcDNAhygro-*RET/PTC1* into FRTL5 cells and established stable lines (FRTL (RET/PTC1) cells). Quantitative RT-PCR using the plasmid DNA as a standard had revealed that (4±0.6)×10^5^ copies/μg RNA were transfected into the cells. When compared with FRTL (pcDNA) cells, FRTL (RET/PTC1) cells were enlarged and flattened even in the presence of TSH, and their cellular borders were obscured regardless of the presence or absence of TSH (Fig. 1D and E). FRTL (RET/PTC1) cells and control cells were stained with hematoxylin; the nuclei of the FRTL (RET/PTC1) cells were irregularly shaped and larger than those of FRTL (pcDNA) cells (Fig. 1C and F). The nuclei of FRTL (RET/PTC1) contained more nucleoli than did those of FRTL (pcDNA) cells (Fig. 1F).

### Effects of RET/PTC1 on FRTL5 cell proliferation and function

**Figure 2:**
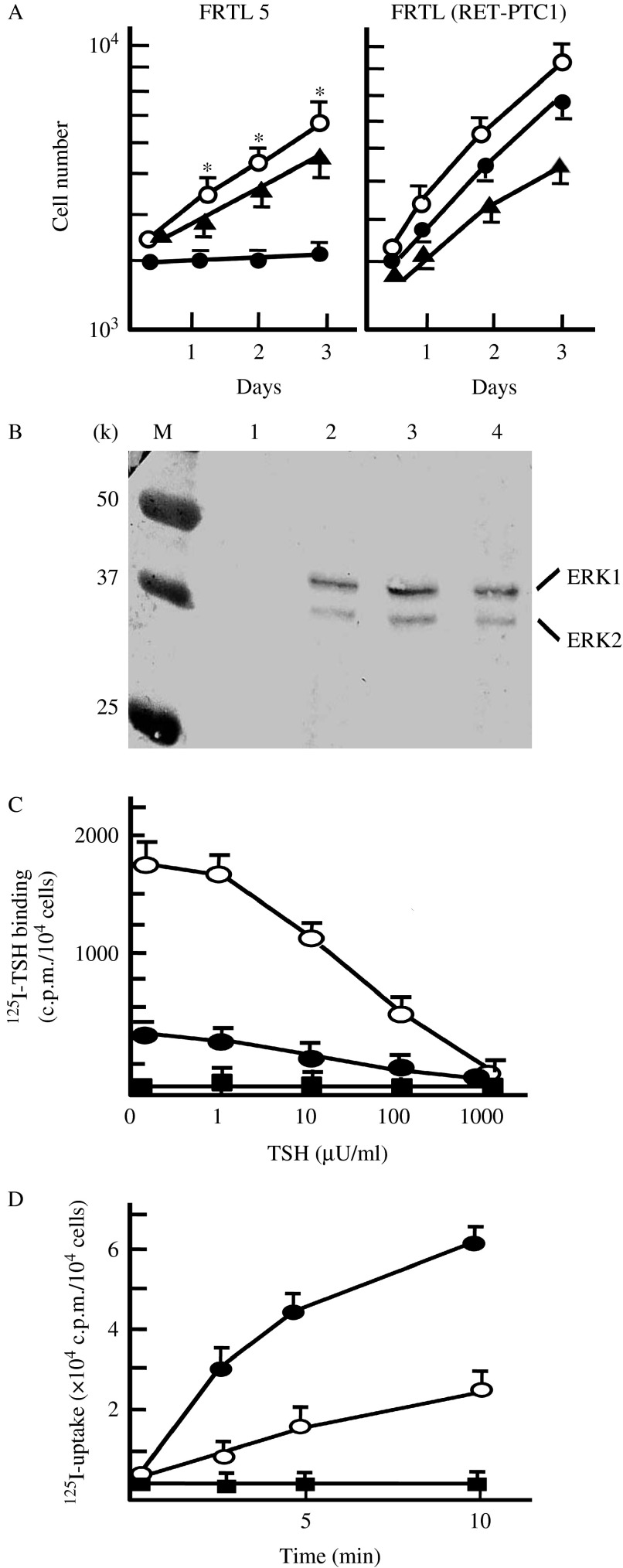
Effects of RET/PTC1 on FRTL5 cell proliferation and function. (A) Growth curves of cultures of FRTL5 cells stably transfected with pcDNAhygro (FRTL (pcDNA)) (FRTL, left panel) or with pcDNA-RET/PTC1 (FRTL (RET/PTC1), right panel) in the presence (open circle–open circle) or absence (closed circle–closed circle) of 1 mU/ml TSH or in the presence of closed triangle–closed triangle: 1 mM dibutyryl cAMP. Data are mean±s.e.m. of three independent experiments. *P<0.01, TSH(+) vs TSH(−). (B) Western blot analysis of ERK phosphorylation. FRTL (pcDNA) cells or FRTL (RET/PTC1) cells were cultured in the absence of TSH for 7 days, and then TSH (1 mU/ml) was added to the culture medium. FRTL (pcDNA) cells before (lane 1) and 3 min after the addition of TSH (lane 2). FRTL (RET/PTC1) cells before (lane 3) and 3 min after the addition of TSH (lane 4). M, molecular weight marker. (C) TSH binding activities of FRTL (RET/PTC1) cells and FRTL (pcDNA) cells. TSH binding activities of FRTL (RET/PTC1) cells (closed circle–closed circle), FRTL (pcDNA) cells (open circle–open circle), or BRL-3A rat liver cells (closed square–closed square) were studied using ^125^I-TSH as a tracer. Data are mean±s.e.m. of triplicate wells of cells. (D) Iodide-uptake activities of FRTL (RET/PTC1) cells (closed circle–closed circle), FRTL (pcDNA) cells (open circle–open circle), or BRL-3A rat liver cells (closed square–closed square). Data are mean±s.e.m. of triplicate wells of cells.

Proliferation of FRTL (pcDNA) cells depends on TSH, and addition of dibutyryl cAMP mimics the effect of TSH (Fig. 2A; Vitti *et al*. 1983). However, there was no significant difference in growth of FRTL (RET/PTC1) cell cultured in the presence or absence of TSH (Fig. 2A). FRTL (RET/PTC1) cells were able to proliferate even in the absence of TSH; the doubling time of the transformed cells was about 18 h, which was shorter than that of FRTL (pcDNA) cells (24 h). Extracellular signal-regulated kinase (ERK) 1 and ERK2 in FRTL (pcDNA) cells were phosphorylated only after the addition of TSH (lane 2), but both proteins were phosphorylated in the presence and in the absence of TSH in FRTL (RET/PTC1) cells (lanes 3–4) (Fig. 2B). FRTL (pcDNA) cells showed high-affinity TSH binding activity (Ka=24 μU/ml). FRTL (RET/PTC1) cells also showed high-affinity TSH binding activity, but total binding activity of FRTL (RET/PTC1) cells was about 30% of that of FRTL (pcDNA) cells (Fig. 2C). In spite of the decreased TSH binding activity, ^125^I-uptake activity of FRTL (RET/PTC1) cells was about 300% of that of FRTL (pcDNA) cells (Fig. 2D).

### Effects of RET/PTC1 on the expression of genes encoding thyroid-specific proteins in FRTL5 cells

We used quantitative RT-PCR to measure the expression of thyroid-specific mRNAs, such as *Tg*, *Nis*, or *Tshr* in FRTL (RET/PTC1) cells. When target-gene expression in FRTL (pcDNA) cells was defined as 1.0, expression of *Tg* and *Nis* mRNAs in FRTL (RET/PTC1) cells was 5.66 and 4.74 respectively; in contrast, expression of *Tshr* mRNAs in FRTL (RET/PTC1) cells was 0.26 (Table 1).

Expression of *Tg* and *Nis* mRNAs in FRTL (RET/PTC1) cells differed significantly from that in FRTL (pcDNA) cells; therefore, we studied the effect of RET/PTC1 on expression of three genes encoding thyroid-specific transcription factors – *Ttf1*, *Ttf2*, and *Pax8* – in FRTL (RET/PTC1) and FRTL (pcDNA) cells. Expression *Ttf1* mRNA was significantly higher (4.68-fold, *P*<0.01) in FRTL (RET/PTC1) cells than in FRTL (pcDNA) cells. However, expression of *Ttf2* and *Pax8* did not differ significantly between FRTL (RET/PTC1) cells and FRTL (pcDNA) cells (Table 1). These results indicated that RET/PTC1 altered the expression level of *Ttf1*, which increased gene expression of *Tg* and *Nis* in FRTL5 cells.

### Tumorigenicity of FRTL (RET/PTC1) cells

**Figure 3:**
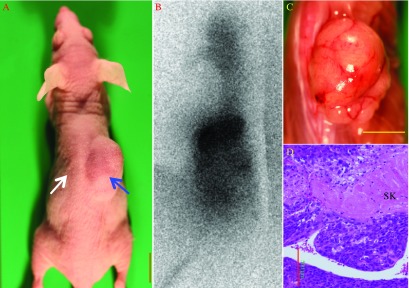
Tumor formation from FRTL (RET/PTC1) cells injected into nude mice. (A) Tumor formation from FRTL (RET/PTC1) cells. FRTL (RET/PTC1) cells (10^6^ cells) or FRTL (pcDNA) cells (10^6^ cells) were transplanted into the subcutaneous tissues of the right (blue arrow) back and the left (white arrow) back respectively of a nude mouse. Two months after transplantation, a tumor had formed on the right side of the back at the (FRTL (RET/PTC1) cell injection site). (B) ^125^I scintigraphy of the tumor. Na^125^I (5×10^6^ c.p.m.) was injected into the peritoneal space, and 6 h later, radioactivity was imaged by exposing the back to an imaging plate. ^125^I had accumulated on the right side back. (C and D) Macroscopic (C, bar: 1 cm) and microscopic (D, hematoxylin–eosin staining; scale bar, 500 μm) features of the tumor derived from FRTL (RET/PTC1) cells. SK, skeletal muscle.

We transplanted FRTL (RET/PTC1) cells into subcutaneous tissues on the right side of the backs of Balb/c nude mice; as controls, FRTL (pcDNA) cells were transplanted into subcutaneous tissues on the left side of the back of each mouse. After 2 months, tumor formation was evident at the site of FRTL (RET/PTC1) cell injection in six of ten mice, but one mouse did not have a tumor form at the site of FRTL (pcDNA) cell injection. By 4 months after injection, each mouse (*n*=9) had developed a tumor at the site of FRTL (RET/PTC1) cell injection, and the mean of the maximum tumor diameter was 24±4.5 mm (Fig. 3A). By contrast, no tumor formation was evident at sites of FRTL (pcDNA) cell injection even 4 months after injection (Table 2). When ^125^I was injected into peritoneal tissues of mice and subsequent scintigraphy was performed at 4 months, accumulation of radioiodide was evident in tumors derived from FRTL (RET/PTC1) cells (Fig. 3B). Figure 3C and D show macroscopic and microscopic views respectively of a tumor derived from injected FRTL (RET/PTC1) cells. Histologically, this solid tumor had a partially glandular structure, and the tumor cells invaded into the surrounding skeletal muscles.

We then further transfected pSilencer (*Ttf1*) into FRTL (RET/PTC1) cells and established stable cell lines (FRTL (RET/PTC1)-si*Ttf1* by selecting with G418. When expression of *Ttf1* in FRTL (pcDNA) cells was set to 1.0, the level of *Ttf1* mRNA in FRTL (RET/PTC1)-si*Ttf1* cells was 0.81±0.042 and not significantly different from that in FRTL (pcDNA) cells.

To investigate the role of TTF1 in tumorigenicity, FRTL (RET/PTC1)-si*Ttf1* cells were injected into the subcutaneous tissues of the backs of nude mice (*n*=9). Even 4 months after injection of the cells, no tumor formation was evident at any of the nine injection sites (Table 2).

### Effects of TTF1 on the human thyroid papillary cancer cell line BHP18-21v

**Figure 4:**
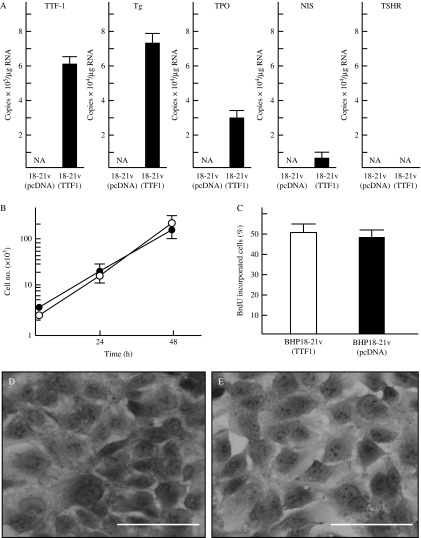
Effects of TTF1 on thyroid-specific gene expression in, cell growth of and, morphology of BHP18-21v cells. (A) Effects of TTF1 on thyroid-specific gene expression in BHP18-21v cells. pcDNAzeo-*hTTF1* was stably transfected into BHP18-21v cells, and expression levels of five thyroid-specific genes (*TTF1*, *TG*, *TPO*, *NIS*, and *TSHR*) in the cells were determined by quantitative PCR. Expression of each gene of interest in human thyroid glands was normalized to that of *GAPDH*, and these values were set as 1.0 for comparisons with expression in BHP18-21v (pcDNA) cells or BHP18-21v (TTF1) cells. NA, no product could be amplified. (B) Growth curves of BHP18-21v (TTF1) (open circle–open circle: *n*=3) cells and of BHP18-21v (pcDNA) cells (closed circle–closed circle: *n*=3). (C) Incorporation of BrdU into BHP18-21v (TTF1) (*n*=3; open column) cells or into BHP18-21v (pcDNA) cells (*n*=3; closed column) 48 h after addition of BrdU. Microscopic views of BHP18-21v (pcDNA) cells (D) or BHP18-21v (TTF1) cells (E). Scale bars, 100 μm.

BHP18-21v cells are derived from a PTC, and they express RET/PTC1. They also express *PAX8* but not *TTF1* or *TG*. Previously, we found that adenovirus-mediated transfer of *Ttf1* into BHP18-21v cells induces re-expression of *TG* and *TPO* (Furuya *et al*. 2004). However, expression of *TTF1* via this method is transient; therefore, we introduced pcDNAzeo-*hTTF1* into BHP18-21v cells to generate BHP18-21v (TTF1) cells that stably express *TTF1*. We then used BHP18-21v (pcDNA) and BHP18-21v (TTF1) cells to study the effect of *TTF1* on tumorigenicity. Quantitative PCR using the plasmid as a standard revealed that (6.2±0.62)×10^5^ copies/μg RNA of *TTF1* (*n*=3) were transfected into BHP18-21v (TTF1) cells. When *TTF1* expression in normal human thyroid glands was defined as 1.0, *TTF1* expression in BHP18-21v (TTF1) cells was 0.84±0.06. Expressions of *TG* and *TPO* genes were undetectable in BHP18-21v (pcDNA), but these genes were re-expressed at (0.51±0.06) (*n*=3) and (0.28±0.03) (*n*=3) respectively in BHP18-21v (TTF1) cells (Fig. 4A). But, *TTF1* showed little effect on *NIS* or *TSHR* gene expression in stably transfected cells as was observed in the transiently transfected cells BHP18-21v (Ad-TTF1) (Furuya *et al*. 2004). There was no significant difference in cell growth (Fig. 4B) or in BrdU incorporation (Fig. 4C) between BHP18-21v (pcDNA) and BHP18-21v (TTF1) cells. *TTF1* also showed little effect on the morphology of BHP18-21v cells (Fig. 4D and E).

**Figure 5:**
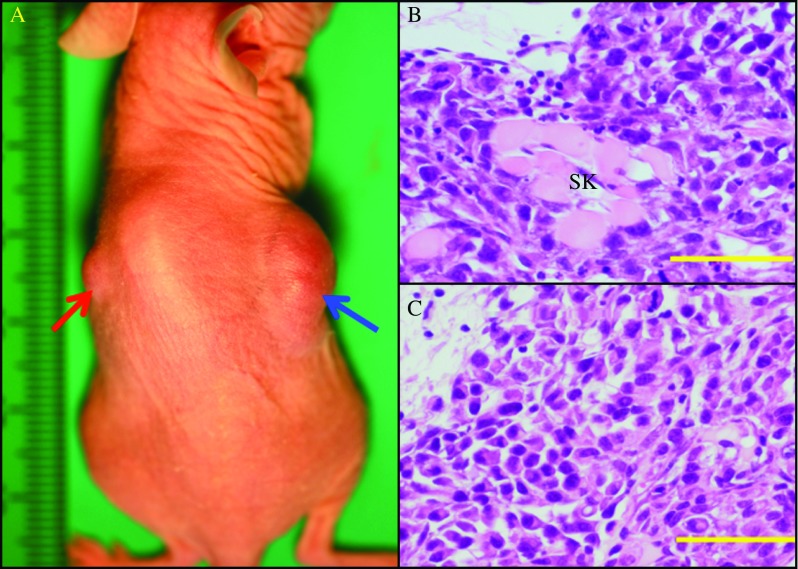
Transplantation of BHP18-21v (TTF1) cells into subcutaneous tissue of nude mice. (A) Macroscopic view of tumors formed by BHP18-21v (TTF1) cells (blue arrow) and by BHP18-21v (pcDNA) cells (red arrow). (B and C) Microscopic views of tumors formed by BHP18-21v (TTF1) cells (B) and by BHP18-21v (pcDNA) cells (C). Scale bars, 20 μm. SK, skeletal muscle.

To investigate the effect of co-expression of TTF1 and RET/PTC1 on the tumorigenicity of BHP18-21v cells, we transplanted BHP18-21v (TTF1) cells into subcutaneous tissues on the right side and BHP18-21v (pcDNA) cells on the left sides of the backs of Balb/c nude mice. After 2 weeks, tumor formation was evident at the site of BHP18-21v (TTF1) cells injection in each of the eight mice, but not one mouse had a tumor form at the site of BHP18-21v (pcDNA) cell injection. After 4 weeks, all mice (*n*=8) had developed a large solid mass at the site of BHP18-21v (TTF1) cell injection, and the mean of the maximum tumor diameter was 25±5.5 mm (Fig. 5A and Table 3). At this 4-week time point, three of the eight mice had developed a tumor at the site of BHP18-21v (pcDNA) cell injection, and the mean of the maximum tumor diameter was 8±2.1 mm for these three tumors. Notably, the mean of the maximum tumor diameter was significantly larger (*P*<0.01) for the BHP18-21v (TTF1) tumors than for the BHP18-21v (pcDNA) tumors. Figure 5A, B, and C shows macroscopic and microscopic views of tumors derived from injected BHP18-21v (TTF1) and BHP18-21v (pcDNA) cells. Histologically, no glandular structure was observed in tumors from BHP18-21v (TTF1) or from BHP18-21v (pcDNA) cells. Notably, BHP18-21v (TTF1) cells had infiltrated into surrounding skeletal muscles within 4 weeks, but BHP18-21v (pcDNA) cells had not.

## Discussion

We used FRTL5 cells for this study because these cells express almost every thyroid-specific gene – including *Tg*, *Nis*, and *Tshr*, as well as the thyroid-specific transcription factors, *Ttf1*, *Ttf2*, and *Pax8* (Santisteban *et al*. 1987). Expression of RET/PTC1 in FRTL5 cells caused nuclei to become irregularly shaped and cell proliferation to become independent of TSH. These changes indicated that expression of RET/PTC1 was sufficient to cause FRTL5 cells to develop malignant phenotypes.

Additionally, De Vita and colleagues expressed RET/PTC1 in PC Cl cells, another line of rat thyroid epithelial cells. *Pax8* expression was significantly lower in PC Cl (RET/PTC1) cells than in the parental PC Cl cells, but TTF1 expression was essentially unaltered by RET/PTC1 expression; function of TTF1 might be inactive (De Vita *et al*. 1998). By contrast, we demonstrated that RET/PTC1 increased the expression of *Ttf1*, *Tg*, and *Nis* in FRTL5 cells, but RET/PTC1 had little or no effect on *Ttf2* and *Pax8* expression. These results indicated that TTF1 functions in FRTL5 cells. Therefore, the results reported by De Vita *et al*. seem to conflict with our findings.

FRTL5 and PC Cl cells present similar sets of properties, including i) TSH-dependent growth and differentiated functions, ii) iodine-uptake, and iii) *Tg* and *Tpo* gene transcription. The transformation of PC Cl cells requires a combination of two retroviral oncogenes, but one oncogene is sufficient to fully transform FRTL5 cells; this difference indicates that FRTL5 cells may intrinsically express some oncogenic function that contribute to a fully malignant phenotype, but PC Cl cells may lack this function (Fusco *et al*. 1987). Although the discrepancy between our results and those of De Vita remains unresolved, it might due to a difference in the precancerous condition of PC Cl cell and that of FRTL5 cells.

TTF1 is a master regulator of the expression of *Tg* and *NIS* (Sinclair *et al*. 1990, Endo *et al*. 1997), and increased levels of *Tg* and *Nis* in FRTL (RET/PTC1) cells might be due to increased expression of TTF1. Indeed, when FRTL (RET/PTC1) cells were transplanted into the subcutaneous tissue of nude mice, the resulting tumor cells retained ^125^I-uptake activity, as do some human papillary thyroid cancer tissues. These findings were consistent with the findings that human PTCs that expressed RET/PTC1 maintained *NIS* gene expression, but those that expressed BRAF^V600E^ did not (Romei *et al*. 2008).

It is of particular interest that FRTL (RET/PTC1) cells were tumorigenic in nude mice, in spite of the fact that FRTL (pcDNA) cells were not. Further, when *TTF1* gene expression was silenced by si*TTF1*, FRTL (RET/PTC1) cells failed to form tumors in nude mice. Similarly, TTF1 was also important to the tumorigenicity of BHP18-21v cells. These cells lacked intrinsic *TTF1* expression; however, stable expression of *TTF1* from a transgenic expression construct increased *TG* and *TPO* mRNA levels in cultures of these cells; moreover, transgenic expression of TTF1 enhanced tumorigenicity of these cells when they were transplanted into nude mice. These results indicated that the oncoprotein, RET/PTC1, and the thyroid-specific transcription factor, TTF1, might interact and consequently affect the tumorigenicity of PTCs.

Therefore, our present results might provide a new insight into the roles of the oncoprotein, RET/PTC1, and thyroid-specific transcription factor, TTF1, in the pathogenesis of PTCs.

## Figures and Tables

**Table 1:** Effects of RET-PTC1 on the expression of thyroid-specific genes in FRTL5 cells

	**FRTL5 (pcDNA)**	**FRTL5 (RET-PTC1)**^a^
*Tg/Gapdh*	1.00	5.66±0.25*
*Nis/Gapdh*	1.00	4.74±0.25*
*Tshr/Gapdh*	1.00	0.26±0.04*
*Ttf1/Gapdh*	1.00	4.68±0.34*
*Ttf2/Gapdh*	1.00	0.97±0.24
*Pax8/Gapdh*	1.00	1.28±0.34

Data are mean±s.e.m. of three independent experiments. *P<0.01.

a Expression of each gene of the interest in FRTL (pcDNA) cells was set as 1.0 for comparison with the respective gene in FRTL5 (RET-PTC1) cells.

**Table 2:** Tumorigenicity of FRTL5 cells in nude mice

	***Ttf1* expression**	**Tumor formation at 2M** (mean of maximum diameter of tumors (mm))	**Tumor formation at 4M** (mean of maximum diameter of tumors (mm))
FRTL5 (pcDNA)	1.0	0/10	0/9
FRTL5 (RET-PTC1)	4.68±0.34*,a	6/10* (8.2±1.1)*	9/9* (24±4.5)*
FRTL5 (RET-PTC1)+pSilenc-TTF1	0.81±0.042^a^	0/9	0/9

Data are mean±s.e.m. of three independent experiments. *P<0.001 vs FRTL5 (pcDNA).

a Expression of *TTF1* mRNA in FRTL (pcDNA) cells was set as 1.0.

**Table 3:** Tumorigenicity of BHP19-21v cell derivatives in nude mice

	**Tumor formation at 2 weeks** (mean of maximum diameter of tumors (mm))	**Tumor formation at 3 weeks** (mean of maximum diameter of tumors (mm))	**Tumor formation at 4 weeks** (mean of maximum diameter of tumors (mm))
BHP18-21v (pcDNA)	0/8	1/8	3/8 (8.1±2.1)
BHP18-21v (TTF1)	8/8* (12.3±2.4)*	8/8* (17.8±3.9)*	8/8* (25.0±5.5)*

*P<0.001 vs BHP18-21v (pcDNA).
